# Genetic Diversity Resonates With Conservation Strategies: A Case Study of *Labeo rohita* Population

**DOI:** 10.1002/ece3.71480

**Published:** 2025-05-25

**Authors:** Md. Mahfuzur Rahman, Khandaker Asif Ahmed, Md. Golam Rabbane, Mohammad Shamimul Alam

**Affiliations:** ^1^ Genetics and Molecular Biology Laboratory, Department of Zoology University of Dhaka Dhaka Bangladesh; ^2^ CSIRO Australian Animal Health Laboratory (AAHL) Australian Centre for Disease Preparedness (ACDP) East Geelong Victoria Australia; ^3^ Fisheries Genetics and Biotechnology Laboratory, Department of Fisheries University of Dhaka Dhaka Bangladesh

**Keywords:** conservation, cytochrome b, genetic diversity, haplotypes, *Labeo rohita*

## Abstract

Conservation strategies often overlook genetic diversity, which is essential for biodiversity preservation and species adaptability. Efforts are minimal for low‐income countries like Bangladesh. The current study assessed the population variances of *Labeo rohita* , a commercially important freshwater fish of Bangladesh, to find the impact of historical policy efforts in conserving diversity. Partial sequences of the mitochondrial *cytochrome b* gene (*cytb*) of 137 samples, originating from three major rivers (Padma, Jamuna, and Halda) and from randomly sampled cultured individuals, were analyzed. Significant differentiation was detected among the groups, with most genetic variation (90.35%) within groups. Eleven haplotypes were identified, including the most frequent haplotype (Hap1BD, 107/137). The geographically isolated Halda subpopulation, with earlier stronger conservation policy, displayed the highest haplotype (0.494) and nucleotide diversities (0.00133) compared to those of the Padma and Jamuna. The culture group also showed distinct diversity and haplotype patterns, which indicate an admixture of *L. rohita* fishes from different sources, including the Halda subpopulation. Pair‐wise FST analysis indicated minimal genetic divergence between the Padma and Jamuna samples (FST = 0.00376), reflecting their geographical connections and moderate conservation strategies. Phylogenetic and haplotype network analyses revealed two distinct genetic clusters, with Jamuna and Padma clustering separately from the Halda subpopulation. Relatively lower effective population size estimation in both Padma and Jamuna could be a reflection of the loose conservation policy on these two rivers. This connection between conservation rules and genetic diversity in the Halda and other rivers indicates that conservation policy efforts, besides many other factors, might impact genetic variability, offering hope for future biodiversity conservation.

## Graphical abstract

The study examined genetic diversity in *Labeo rohita* from three major rivers and a cultured assemblage of Bangladesh to find its correlation with conservation policies. The Halda subpopulation, helped by relatively stricter conservation efforts, had the most genetic diversity, whereas the Padma and Jamuna collections had decreasing diversity and effective population sizes. These findings highlight the importance of conservation measures in preserving genetic variability and biodiversity.

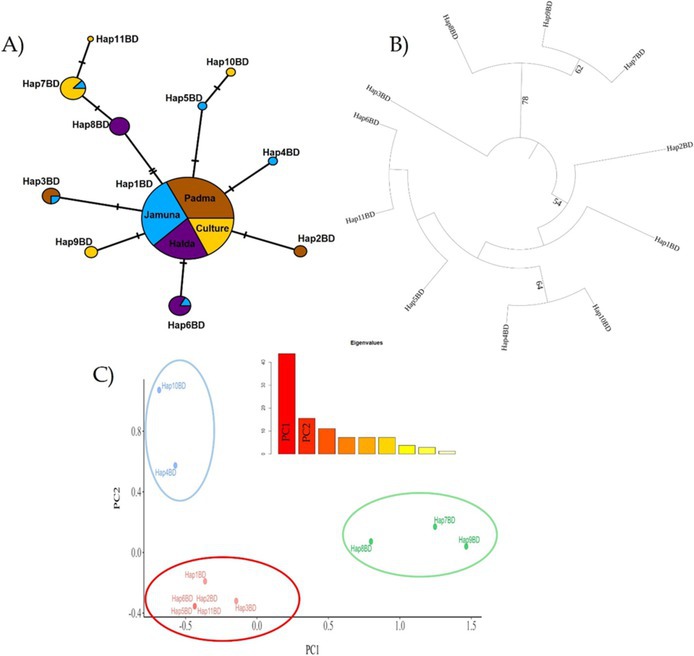

## 1 Introduction

Genetic diversity is a basic component of biodiversity and is essential for the adaptability and long‐term survival of a species (DeWoody et al. 2021). Reduced genetic diversity in threatened species can lead to inbreeding depression and reduction in reproductive and adaptive fitness, further endangering populations (Willoughby et al. 2015). Global conservation policies have often overlooked genetic diversity (Hoban et al. 2020). The Convention on Biological Diversity (CBD) has emphasized maintaining and restoring the genetic diversity of species populations (CBD 2022). Advances in technology and data availability make it feasible to integrate genetic diversity into policy frameworks (Hoban et al. 2021). In low‐ and middle‐income countries like Bangladesh, data on genetic resources for monitoring population diversity are scarce, although some conservation strategies for maintenance and preservation exist.

Bangladesh, a country with vast river networks, has implemented various conservation policies to protect and preserve its aquatic biodiversity. While the rules and regulations for aquatic conservation have primarily focused on overall fisheries resources, specific measures have been implemented to safeguard and conserve the native carp species found in specific rivers. These measures include fishing prohibitions for certain periods or year‐round. In the case of the Halda River, a prohibition on catching the carp fish from 15th March to 30th June (three and half months) each year was established by the Protection and Conservation of Fish Rules, 1985 (The Bangladesh Gazette 1985a). These rules have been amended occasionally to protect and conserve the fish species. In 2007, a 20‐km stretch of the Halda River crucial for breeding and spawning was declared a fish sanctuary with a total fishing prohibition throughout the year. These interventions have been in effect till now. Historically, as a popular carp fish breeding ground in Bangladesh, the Halda River has been subjected to stricter conservation rules since 2007 (MoFL, 2007).

The Protection and Conservation of Fish Rules, 1985, prohibit catching carp fish in certain regions of the Jamuna River for 4 months (April–July) each year (The Bangladesh Gazette 1985b). Later, in 2021, major carp fish breeding areas of the Jamuna River were declared as fish sanctuaries, imposing year‐round fishing restrictions. In the case of the Padma River, a 20‐km stretch of the river was subjected to a 2‐month fishing ban each year since 2011 (March–April) (MoFL, 2011). This rule was mainly targeted at the protection of the Hilsha fish. The prohibition during this period might not significantly impact carp fish due to its little congruence with carp fish conservation in terms of time and space. As many conservation rules and laws were implemented at different scales and times, it is important to observe if there is any association between conservation strategies and genetic diversity and how it can be improved for the betterment of economically important fish species.

One of Bangladesh's economically important freshwater fish species is *Labeo rohita* (Hamilton, 1822), commonly known as Rohu (Behera et al. 2018). It is also common in other South Asian countries, including India, Pakistan, Nepal, and Myanmar. Belonging to the family Cyprinidae, *L. rohita* offers significant nutritional benefits and is widely cultivated in freshwater bodies due to its fast growth, delicious taste, and high market demand (Behera et al. 2007; Hussain and Mazid 2005). Its contribution (including capture and Culture) to the overall aquaculture production of Bangladesh increased from 8.83% in 2005 (Rahman 2005) to 11.56% in 2023 (DoF 2023a). The amount of cultured rohu fish in closed water bodies (e.g., ponds) is 22,72,667 metric tons, which is 53.66% of total rohu fish production (DoF 2023b). The culture is dependent on isolated hatchery sources. So, there is a high chance of occurring inbreeding, which can result in retardation in growth, development, and reproduction, decreased fitness in offspring, and reduction in yield (Neaves et al. 2015; Piles et al. 2022). Identifying genetically diversified wild populations and outcrossing can minimize such depression.

The wild fishes of *L. rohita* are sourced from different aquatic bodies, including rivers, lakes, back swamps (haors), oxbow lakes (baors), etc. The Halda, Jamuna, and Padma rivers are the three important rivers from where wild fishes and their eggs/hatchlings are collected. The Halda is well‐known as the natural breeding ground of carp fishes, and it has recently been declared a fisheries heritage site (Halda Gazette 2020). Its unique tidal nature and oxbow bends make it a suitable place for carps to release eggs. The Halda River is geographically distant from the Jamuna and Padma rivers and is not connected to any of these. The Jamuna River flows southward and joins the Padma River near Goalanda Ghat. Early studies designated the fishes of each river as independent populations based on their geographical location (Alam et al. 2009; Islam and Alam 2004). Although genetic lineage has not been determined yet, the genetic diversity of the *L. rohita* population inhabiting these rivers and culture facilities has been studied previously using different molecular markers.

Islam and Alam (2004) conducted a study to evaluate the genetic variation in populations of *L. rohita* from three rivers (Halda, Jamuna, and Padma) and one hatchery in Bangladesh using RAPD markers. Their study revealed low or no significant genetic differentiation among the population pairs. In the UPGMA dendrogram on Nei's genetic distance, the Halda population formed one group, while the Jamuna, Padma, and hatchery fishes formed another group (Islam and Alam 2004). However, a later study conducted by Alam et al. (2009) found significant population differentiation between the Halda and Jamuna populations using microsatellite DNA markers known for their higher resolution and greater sensitivity to genetic variation. Consistent with the earlier study, Halda was found to be distinct from the Jamuna and Padma populations in the UPGMA dendrogram. The distinct grouping of the Halda population could be attributed to its unique geographical location, river basin structure, and historical conservation policy factors that have led to genetic differentiation from the other populations. Recently, mitochondrial genes, especially the partial *cytb* gene—have been widely used to compare genetic structures of several *L. rohita* (Ahammad et al. 2022; Behera et al. 2018; Luhariya et al. 2012; Sarmah et al. 2022) and other fish species (Bouza et al. 2008; Murray et al. 2008; Oleinik et al. 2007; Tagliavini et al. 1999). There is a scarcity of *cytb* gene sequences of the *L. rohita* population, harvested across major rivers of Bangladesh, which can broaden our understanding of ongoing conservation strategies across these rivers and how diverse culture stocks are compared to the riverine fishes.

The current study aims to examine the genetic diversity of river‐origin and hatchery populations of *L. rohita* based on the mitochondrial *cytb* gene region and discuss the association between fish genetic diversity and conservation policy efforts in the respective wild habitats of Bangladesh. The findings could assist in formulating conservation strategies for wild populations of *L. rohita* fish and provide insights into the preservation of genetic variety, ultimately leading to enhanced productivity.

## 2 Materials and Methods

### 2.1 Study Area and Sampling

**FIGURE 1:**
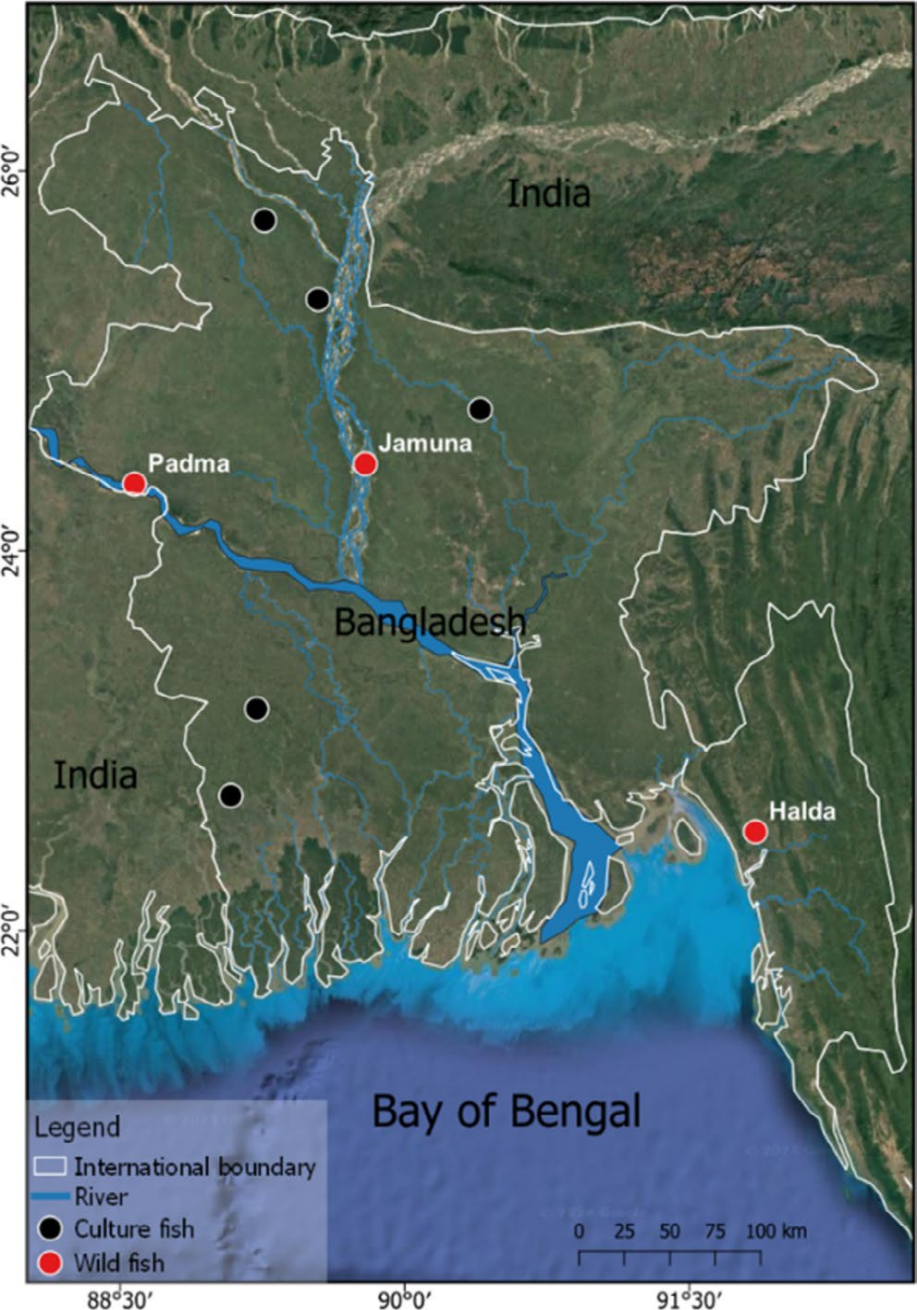
*L. rohita* sample collection sites are shown on the map of Bangladesh.

From 2021 to 2023, Rohu fish samples were collected from wild and cultured sources. Due to the prohibition on direct fishing in the Halda and Jamuna rivers and scarcity in Padma, wild fish samples were collected from regional fish farmers who collected fish fries or eggs from respective rivers and raised those in their ponds separately. Thus, 150 river‐origin dead, fresh fish samples were collected from the fish farmers located near Halda, Padma, and Jamuna rivers. Fifty fresh samples were commercially sourced from the local markets of Jessore, Satkhira, Gaibandha, Rangpur, and Mymensingh and verbally confirmed to be from cultured sources (Figure 1). Collected samples were primarily preserved in ice boxes to prevent deterioration during transport from the field to the laboratory. Then, all sampled individuals were identified morphologically (Rahman 2005). The samples were stored at −20°C.

### 2.2 DNA Extraction, Amplification of *Cytb* Gene, and Sequencing

Genomic DNA was extracted using a modified CTAB‐extraction protocol described elsewhere (Siddika et al. 2024). In brief, < 50 mg of fin tissue was homogenized in CTAB buffer and subjected to degradation of residual proteins and RNA using Proteinase K (Sigma Aldrich, Germany) and RNAse (Carl Roth, Germany), respectively (Alam et al. 2016; Begum et al. 2019). After the removal of cellular debris through centrifugation, a mixture of phenol‐chloroform‐isoamyl alcohol in the ratio of 25:24:1 was added to the supernatant in equal volume. DNA was isolated through ethanol precipitation, dried in air, and dissolved in TE buffer. DNA integrity and relative concentration were checked in 1% agarose gel electrophoresis. To amplify the *cytb* gene of *L. rohita* , two sets of primers were designed using NCBI Primer Blast, with default parameters and an expected product size of 800–900 bp. The primers (Macrogen, Korea) were checked for nontarget PCR co‐amplification. Based on PCR product size, nontarget co‐amplification was not detected in the gel after electrophoresis. One pair of primers was chosen to amplify the target region of *cytb*. The primer details are shown in Table 1. The PCR reactions were conducted in a 20 μL volume using a PCR master mix (Takara Bio, Shiga, Japan). The thermal cycling profile was set as follows: initial denaturation at 95°C for 5 min, followed by 35 cycles of denaturation at 95°C for 30 s, annealing at 61°C for 30 s, and elongation at 72°C for 60 s. A final elongation step was performed at 72°C for 7 min. The PCR products were purified using the PCR purification kit (US Everbright, China). A total of 200 samples (50 from each location) were sent to Macrogen Inc. (Korea) for capillary sequencing in both directions. However, due to sequence quality and length variations, the *cytb* sequence from 137 fish samples was further analyzed.

**TABLE 1:** The forward and reverse primers used to amplify a specific region of the mitochondrial *cytb* gene.

Primer name	Nucleotide length	GC%	Sequence	Product length	Annealing temperature
CYTB‐F	20	45	AACCGAGACCAATGACTTGA	828	61°C
CYTB‐R	20	50	GGGTGAAGTTTTCTGGGTCT		

### 2.3 Sequence Data Analysis

The chromatograph quality of the raw sequences was assessed, forward and reverse reads were aligned, and quality base readings were extracted into distinct fasta files using Finch TV software version 1.4.0 (http://www.geospiza.com/Products/finchtv.shtml). The amplified fragment's origin was confirmed using NCBI's BLASTn search program. The ClustalW algorithm (Thompson et al. 1994) within MEGA v.11 (Tamura et al. 2021) and BioEdit 7.1.3.0 (Hall 1999) software programs were used to accomplish multiple sequence alignment. The sequencing data were examined to ascertain the sequence composition, count the number of polymorphic sites, identify parsimony informative sites, and apply the maximum‐likelihood (ML) and neighbor‐joining (NJ) topology approaches with 1000 bootstrap replicates (Felsenstein 1985; Tamura et al. 2021). The sequences were subsequently allocated to haplotypes.

The dataset was used to construct haplotype networks using NETWORK 4.6.1.2 (Bandelt et al. 1999). The haplotype and nucleotide diversities were estimated using DnaSP v. 5.1 (Librado and Rozas 2009). Principal component analysis was conducted on 11 haplotypes. Adegent R package was used to convert fasta files to genlight format, followed by eigenvalue estimation using SNP variations (Jombart 2008; Jombart and Ahmed 2011). PC1 and PC2 were plotted using ggplot2 (Wickham et al. 2019) and ggrepel R packages to get the final PCA plot (Slowikowski 2024).

A priori test of genetic lineage number (k) was conducted in Geneland R Package. Genetic variation within and among the sampled groups, genetic differentiation, and pairwise Fst values were computed to analyze molecular variance (AMOVA) using Arlequin version 3.5 (Excoffier and Lischer 2010). To evaluate the association between haplotypes and their geographical patterns, a minimum‐spanning tree was constructed using PopArt version 0.6 (Griffiths et al. 2010).

Effective population sizes (N_e_) for each subgroup, Padma, Jamuna, Halda, cultured, and all combined were calculated using the Coalescent formula (Hudson 1991; Kingman 1982; Tajima 1989). The individual sequences within each subgroup were aligned using the MUSCLE program within MEGA v11(Tamura et al. 2021), followed by overall mean computation, with 1000 bootstrap replicons, nucleotide substitution model, and p‐distance method, which generated the standard error estimates of Nucleotide diversity (SE of Pi) for a given population. Theta and effective population sizes were estimated using the following formula:

$$\text{theta}\;\left(\theta \right)=\mathrm{pi}*n/\left(n-1\right),$$

$$\text{Effective population size}\;\left(\mathrm{Ne}\right)=\theta /\left(4*\mathrm{mu}\right),$$

$$S.E\;\text{of}\;N_{e}=\mathrm{SE}\;\text{of}\;\mathrm{Pi}/\left(4*\mathrm{mu}\right)$$

Where, *n* = sequence length considered (616 bp), pi = nucleotide diversity for a given population, SE of pi = standard error of nucleotide diversity, mu = 3.11e −8, which had been identified as the cytb mutation rate in Salmonoid fishes (Shedko 2019). These data were plotted using the ggplot2 R package (Wickham et al. 2019).

## 3 Results

Based on the chromatograms of forward and reverse sequences of amplified products, a stretch of the *cytb* gene (616 bp long) has been analyzed further.

### 3.1 Haplotype Diversity and Population‐Specific Variations

We thoroughly analyzed 137 samples from four different fish groups to estimate individual subpopulation structures and haplotype compositions (Table 2). Even though we analyzed only a 616 bp region of the *cytb* gene, with > 606 bp monomer sites in all four groups, both Jamuna and Cultured samples had seven, and Padma and Halda had two and three polymorphic sites, respectively. In terms of haplotype and nucleotide diversity, the cultured population showed the highest diversities (0.539 and 0.0024), whereas the lowest diversities were seen in the Padma population (0.232 and 00039). While no parsimony sites were observed in the studied *cytb* region of the Padma and Jamuna populations, three and four parsimony sites were still found in the Halda and cultured populations.

**TABLE 2:** Diversity analysis at mitochondrial *cytb* locus of four populations.

Population	Padma	Jamuna	Halda	Culture	All
No of individual	40	36	32	29	137
No of Haplotype	3	6	3	5	11
Haplotype diversity	0.232	0.262	0.494	0.539	0.385
Nucleotide diversity	0.00039	0.00063	0.00133	0.0024	0.00118
Monomorphic sites	614	609	613	609	606
Polymorphic sites	2	7	3	7	10
Singleton sites	2	7	0	3	2
Parsimony sites	0	0	3	4	8

**FIGURE 2:**
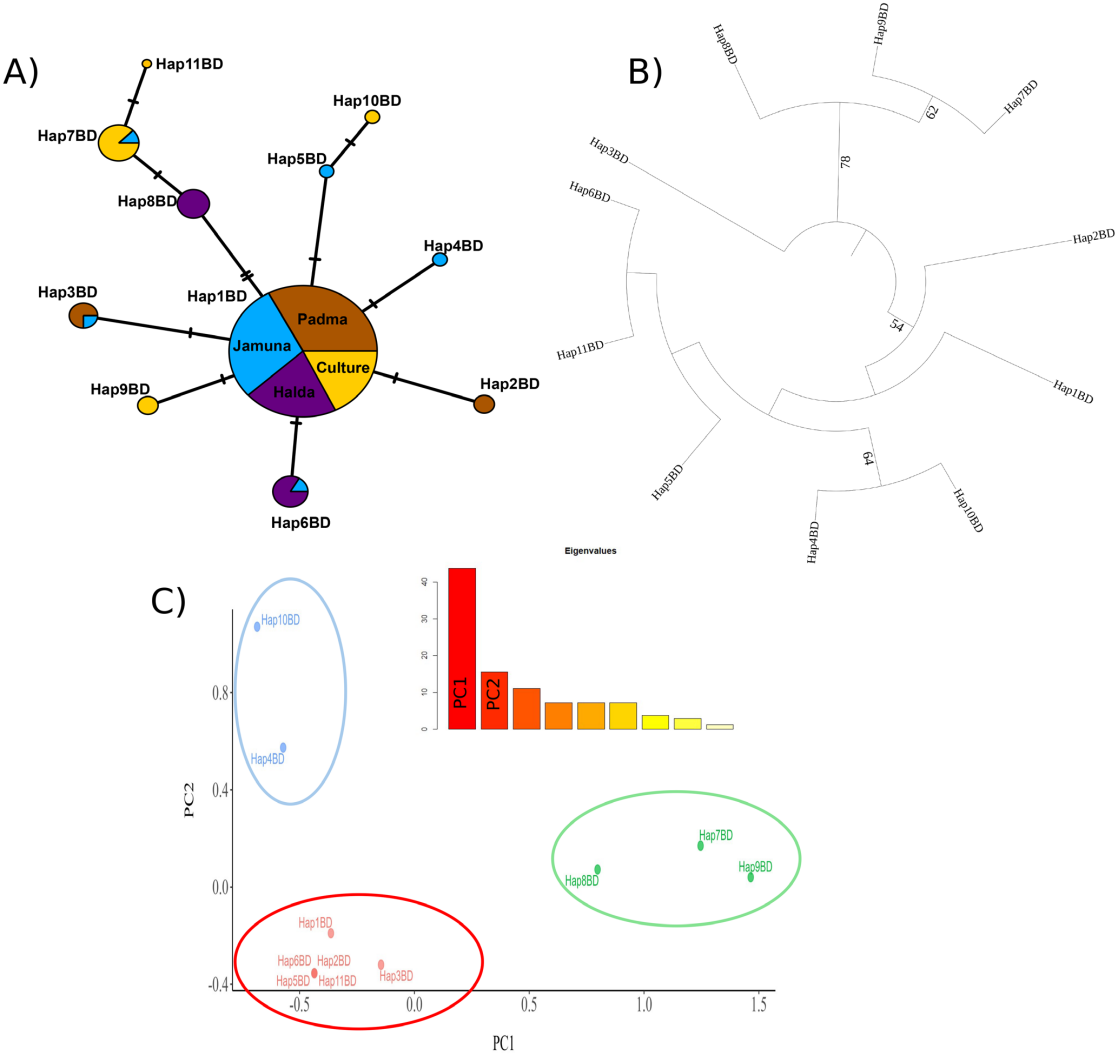
Haplotype diversity in ***L. rohita***. (A) Haplotype network diversity, (B) Maximum likelihood tree, with 1000 bootstrap replicates; bootstrap percentage supports (> 50%) are shown in different nodes, (C) Principal component analysis (PCA) and plotting PC1 and PC2—which covers > 60% variances of all haplotypes.

We identified 11 haplotypes (Accession nos. PQ458546–PQ458556) across the four subpopulations. The Jamuna collection had the highest number of haplotypes (*N* = 6), followed by Culture (*N* = 5), Padma (*N* = 3), and Halda (*N* = 3). The overall percentages of different haplotypes have been shown in File S1, and their diversity was evaluated using different metrics in Figure 2. The Hap1BD haplotype was the most frequent, observed in 107 samples, covering 78% of the whole sampling. The other haplotypes are represented in a very low percentage, such as Hap3BD (3%), Hap6BD (4.5%), Hap7BD (5.1%), Hap8D (3.64%), etc. Among the groups, the haplotype Hap1BD was found in 88% of individuals of the Padma River, 86% in Jamuna, 72% in Halda, and 66% in the culture. Notably, group‐specific haplotypes were observed. For instance, the culture group has three distinct haplotypes (Hap9BD, Hap10BD, and Hap11BD) that were absent in other groups: the Halda one (Hap8BD), the Jamuna two (Hap4BD and Hap5BD), and the Padma one (Hap2BD). Furthermore, the study identified shared haplotypes between populations. Hap3BD was common between Jamuna and Padma groups, Hap6BD between Halda and Jamuna, and Hap7BD between culture and Jamuna. Regarding base‐pair variations, Hap2BD, Hap3BD, Hap4BD, Hap6BD, and Hap9BD each exhibit a single nucleotide substitution from the most common haplotype Hap1BD. Hap8BD differs by two substitutions from Hap1BD, while Hap7BD diverges by one substitution from Hap8BD and three substitutions from Hap1BD. Lastly, Hap11BD displays four substitutions relative to Hap1BD (File S2).

This study analyzed haplotype diversity in the Bangladeshi population, focusing on distinct haplotypes' distribution and unique characteristics. Figure 2B shows the phylogenetic tree constructed using all 11 haplotypes (Hap1BD–Hap11BD). The tree shows a clear cluster among different haplotypes. The tree topology indicates the evolutionary relationships among these haplotypes, but with low bootstrap support, which is possibly due to the minimum differences between haplotypes. Bootstrap values provided at certain nodes indicate the statistical support (only > 50% considered) for the corresponding clades. Notably, Hap4BD, Hap5BD, Hap6BD, Hap10BD, and Hap11BD form a well‐supported clade, indicating close evolutionary relationships among these haplotypes. Hap7BD, Hap8BD, and Hap9BD showed common ancestry and diversification from other haplotypes. In contrast, other haplotypes, such as Hap1BD, Hap2BD, and Hap3BD display more basal positions, suggesting earlier divergence from the common ancestor of the clade.

### 3.2 Genetic Lineage and Analysis of Molecular Variance (AMOVA)

Before conducting AMOVA, we conducted an a priori test to identify the potential number of genetic lineages within the collection groups. The analysis was conducted on both spatial and non‐spatial modes, but results were similar (File S3). We found genetic lineage number, k = 1, which indicates all individuals belong to a single population. There is no strong genetic differentiation among the individuals collected from different rivers and cultures. The analysis of molecular variance (AMOVA) was conducted to assess the distribution of genetic variation among and within the collection sites (Table 3). The overall fixation index was 0.965, which indicates complete differentiation among the four groups. Further, it also showed that a significant portion of the genetic variation, 9.65%, is attributed to differences among the groups collected from separate rivers and cultures (df = 3; SS = 4.69; *p* = 0.0). Most genetic variations (90.35%) are found within the four sites (df = 133; SS = 44.826; *p* = 0.0). The variance components (Va, Vb) associated with among and within sites are 0.03600 and 0.33704, respectively, reflecting the extent of genetic diversity in each collection site. These results suggest that even though the individuals collected from different locations belong to the same population, individual sites exhibit substantial genetic variability, contributing significantly to the overall genetic makeup of the overall fish population.

**TABLE 3:** AMOVA analysis of *L rohita* based on *cytb* sequence (FST = 0.965; *p*‐value = 0.0).

Source of variation	df	Sum of square	Variance of component	Percentage of variation
Among groups	3	4.692	0.03600^Va^	9.65
Within groups	133	44.826	0.33704^Vb^	90.35
Total	136	49.518	0.37304	

The genetic differentiation among four distinct sites of *L. rohita* was assessed using pairwise FST values in Table 4. Among all the pairs, Padma and Jamuna showed the lowest FST value (0.00376) with no statistical significance at a 5% level. The FST value between Culture and Halda sites (FST = 0.05701) was also low without statistical significance. These numbers indicate less genetic variability between Padma and Jamuna, and Halda and Culture sites. However, each of the Padma and Jamuna sample groups was significantly divergent from the Halda and culture group separately at a 5% level. Especially, the highest difference was found between Padma and Culture (Fst = 0.1764, *p* < 0.01), which indicates these two groups are substantially differentiated. An almost similar scenario was observed between Padma and Halda (Fst = 0.11987, *p* < 0.01), and Jamuna and culture (Fst = 0.11845, *p* < 0.01), which indicates some lesser degrees of fixation.

**TABLE 4:** Pairwise FST values among the groups of *L. rohita* population.

	Padma	Jamuna	Halda	Culture
Padma				
Jamuna	0.00376^ns^			
Halda	0.11987^a^	0.05632^a^		
Culture	0.17764^a^	0.11845^a^	0.05701^b^	

^ns^ Not significant.

^a^ *p* < 0.01.

^b^ *p* < 0.07.

**FIGURE 3:**
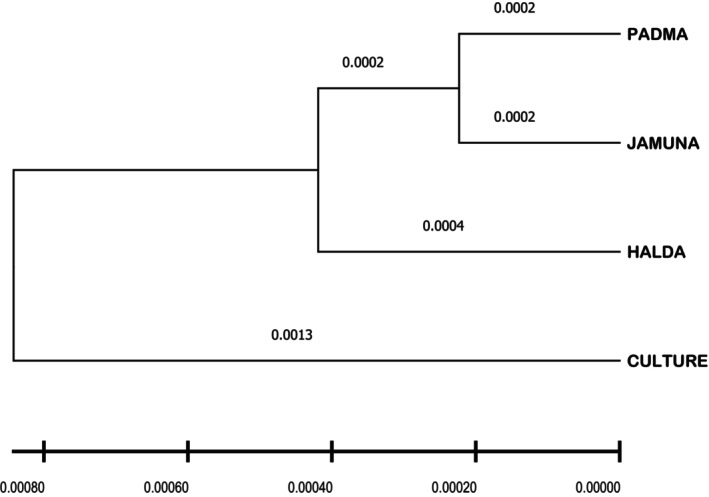
The phylogenetic tree using the Neighbor‐Joining method of *L. rohita* based on the *cytb* sequences.

The UPGMA tree (Figure 3) revealed two genetically distinct clusters: one comprising all wild groups and another consisting solely of the culture group, indicating clear genetic separation between wild and cultured individuals. The first cluster was further separated into two subgroups: the Halda in one group and the Padma and Jamuna in a separate group. The pairwise Fst comparison reconfirms our findings, where Padma and Jamuna, and Halda and culture groups showed less variability. It further validated the highest genetic distance value between Padma and the culture group (0.0013).

**FIGURE 4:**
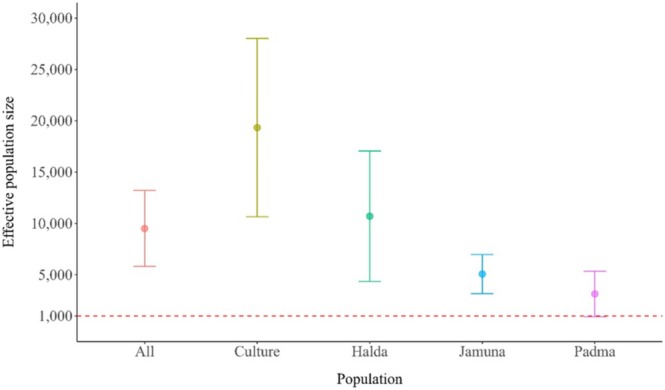
Effective population size (N_e_) estimates for different groups of *L. rohita*. Standard error bars have been shown. The horizontal line is drawn at N_e_ = 1000, which is proposed to be a lower threshold for population adaptability (Frankham et al. 2014).

Finally, we conducted the effective population size (N_e_) estimation (Figure 4). Overall, we identified a population estimate of 9500.95 ±3697.75 across all four groups. While we calculated the estimate individually, we found the highest values in the culture (19323.97±8681.67), followed by Halda (10708.70±6350.48). The lowest estimate was identified in the Padma and then at Jamuna. The large standard error bars also indicate the wide variability in culture and Halda collections, which are narrower in Jamuna and Padma. Interestingly, both Padma and Jamuna groups are approaching the borderline of (N_e_)1000, which has been proposed as the effective population size for maintaining biological diversity and adaptability within a population (Frankham et al. 2014).

## 4 Discussion

The Halda River in Bangladesh is globally recognized as the only natural source of fertilized eggs for Indian major carps, a distinction attributed to its unique hydrological, physicochemical, and ecological conditions (Hossain et al. 2022; Hossen and Jishan 2018). Favorable environmental parameters—such as optimal temperature, turbidity, and dissolved oxygen levels—along with seasonal variations, create an ideal habitat for spawning and larval development in this river (Akther et al. 2023; Islam et al. 2022). Additionally, the river's rich biodiversity, characterized by abundant phytoplankton and zooplankton communities, and its tidal dynamics further support its suitability as a natural breeding ground (Hossen and Jishan 2018; Mojumder et al. 2020).

The historical timeline of fish conservation and protection regulations indicates that the Halda River, a crucial location for carp fish spawning, has been under a year‐round fishing ban since 2007. In contrast, the Jamuna and Padma Rivers did not have such a strict ban until the current research began in 2021. Among the wild habitats, the Halda has been the best breeding ground for carp fish in Bangladesh, and its fish have exhibited higher genetic diversity in almost all previous and present reports (Alam et al. 2009; Islam and Alam 2004; Tonny et al. 2014). There might be many causes for these differences among the rivers, starting from river basin structure, water quality, water flow, river management, and conservation policies, to the societal and economic status of the local people. However, the analysis of the genetic diversity studies published after 2007, including the present one, reveals that the genetic distances between Halda and Padma, and Halda and Jamuna have increased over time, regardless of the overall decline of genetic variability in the *L. rohita* populations of Bangladesh (Alam et al. 2009; Islam and Alam 2004; Tonny et al. 2014).

The current study analyzed the genetic structure of three riverine and one hatchery‐origin cultured group of *L. rohita* . AMOVA results revealed that within‐group variation was higher (90.35% in proportion) compared to that in among‐group variation (9.65%), which coincides with the earlier reports on the *L. rohita* populations from India (Das et al. 2012; Das et al. 2018; Hamilton et al. 2019; Joshi et al. 2019; Sahoo et al. 2019) and is possible because of the increased gene flow among the populations (Allendorf et al. 2012; Slatkin and Barton 1989). Barman et al. (2003) also observed a high level of within‐species genetic similarity (97.5%), that is, low levels of within‐species genetic variation in farmed stocks of major carp in India. This gene flow may lower genetic differentiation (Ferguson et al. 1995). The fixation index (Fst = 0.0965; *p* = 0.0) revealed little genetic variation among the groups, which again aligns with other previous studies (Excoffier et al. 1992; Weir and Cockerham 1984). Generally, individuals with greater genetic variability have higher growth rates, developmental stability, viability, fecundity, and resistance to environmental stress and diseases (Carvalho 1993).

The genetic diversity analysis demonstrated varying levels of haplotype and nucleotide diversities among riverine and cultured groups. Among 137 individuals tested, our study has identified 11 haplotypes, where one Hap1BD covered 78% of the total studied. This haplotype could be the oldest mitochondrial lineage from which all haplotypes originated independently through mutations (Charlesworth 2009). The inclusion of more samples may increase the number of haplotypes. Conservation of unique haplotypes should be sustained to increase diversity. Among the three wild and one cultured group, the cultured samples surprisingly displayed the highest haplotype and nucleotide diversity among all groups, followed by Halda. However, the haplotype and nucleotide diversities we obtained, at 0.385 and 0.00118, respectively, fall within the range of freshwater fish diversity studies across various regions of Bangladesh and India in the past years (Grant and Bowen 1998; Das et al. 2018; Joshi et al. 2019; Sah et al. 2011; Das et al. 2014; Pakrashi et al. 2015; Habib and Vindhya 2011; Luhariya et al. 2012).

We identified a potential genetic lineage number of one, which reveals that all individuals collected from different places in the present study belong to a single homogeneous population. However, previous studies from the same collection sites (Padma and Jamuna—Alam et al. 2009; Halda and Padma—Islam and Alam 2004) mentioned these as distinct populations without a priori testing. Our results suggest potential genetic homogenization within the overall samples but collection‐site‐specific distinct signatures. Among the four studied groups, low‐to‐moderate levels of genetic differentiation were documented (pairwise Fst values ranging from 0.00376 to 0.17764). A similar observation was made by Das et al. (2012) using mtDNA markers; low SNP marker diversity and lack of genetic differentiation were reported by Hamilton et al. (2019). The maximum genetic difference (Fst = 0.17764) between the Padma and culture, and the least genetic difference (0.00376) between the Padma and Jamuna collections indicate relatively high levels of gene flow between Padma and Jamuna, which is again supported by an earlier study (Alam et al. 2009). Smaller genetic distance and high gene flow might be the possible reasons for reduced genetic differentiation between these site‐specific collections. However, each of the Padma and Jamuna groups was significantly divergent from that of the Halda and culture separately at the 5% level. Islam and Alam (2004) found similar results in their RAPD‐based study, where the highest and lowest genetic differences were observed between the Halda and Padma and the Padma and cultured groups, but none of these differences were significant. Similar results were also shown in the microsatellite DNA marker‐based study (Alam et al. 2009), which revealed significant differentiation (Fst) between the Halda and the Jamuna groups. Notably, the pairwise Fst values generated in the present study may not be comparable with those in past studies, which used different DNA markers. Even though these previous works did not allow us to measure genetic distances increased over time, we can still observe similar “relative” genetic distances among the studied groups. However, the present data could be utilized well in future monitoring of the *L. rohita* populations.

Our study also identified a higher genetic distance between the Halda and the Jamuna (Fst =0.05632, *p* < 0.01) or between the Halda and the Padma (Fst = 0.11987, *p* < 0.01). Geographically, Padma is connected to Jamuna, near the Goalanda upazila of the Rajbari district, whereas the southeastern Halda River is distant from the other two (Akter and Ali 2012). A possible mixing between Padma and Jamuna strains cannot be excluded. The presence of a geographical barrier between the Padma or Jamuna and Halda can explain the higher genetic distances between them. The Fst value indicates no significant genetic differentiation between the culture and Halda. The lower genetic distance (Fst = 0.05701, *p* = 0.063) between the Halda and culture suggests that culture stocks may have predominantly originated from the Halda River. However, the presence of unique haplotypes in the culture indicates the possibility of additional sources, such as other rivers, haors, beels, etc. The higher haplotype and nucleotide diversity in the cultured group compared to other wild groups further supports the idea that culture stocks could represent a mixture of different sources.

In 2004, genetic diversity measures revealed low genetic differentiation among the three river populations of *L. rohita* (Islam and Alam 2004). Subsequent studies conducted after 2007 reported increased differences among the groups, with the Halda River gaining stricter policy measures compared to the two other rivers (Alam et al. 2009; Tonny et al. 2014). The lack of stricter and specific conservation policy intervention for *L. rohita* in the Padma River may have contributed to a notable increase in genetic distance between the Halda and Padma River groups in 2014 compared to 2009. In the present study, the genetic distance between Halda and Padma rivers, as indicated by the pairwise Fst value, is the highest (0.119, significant) among the river pairs, which may reflect the conservation policy gaps between these two rivers. Similarly, the Halda and Jamuna subpopulations also differentiate significantly from each other. Thus, the population genetic data correspond to the intensity of previous fishing interventions. However, policy efforts alone are insufficient for conserving biodiversity and genetic diversity.

The impact of legal frameworks on biodiversity depends on how effectively they are implemented. There are challenges in implementing a total fishing ban on a river associated with the livelihood of local people (Azadi and Arshad‐ul‐Alam 2011). It is important to apply strict conservation rules where anthropogenic pressures are high. Factors such as habitat degradation and destruction due to water pollution, encroachment, riverine transport and siltation, unauthorized fishing during the prohibited period, lack of awareness among stakeholders, absence of alternative livelihood options, and natural disasters may also have their impact. Research data on these factors concerning the three major rivers of the present study are largely unavailable. Still, the chronological analysis of the previous and present population genetic structure of *L. rohita* and policy efforts in the respective rivers suggests that the appropriate conservation policies with proper implementation might positively impact the conservation of population diversity.

The effective population size (N_e_) estimates provide important insights into the genetic stability of the populations. Even though all individuals belong to the same population, the site‐specific estimates indicated some valuable insights. The cultured group exhibited the highest N_e_, followed by Halda, reflecting their greater genetic diversity. In contrast, the Padma and Jamuna subpopulations showed lower N_e_ values, both approaching the critical threshold of 1000 individuals, which may threaten the long‐term genetic diversity and adaptability of the species (Frankham et al. 2014). The smaller N_e_ in Padma and Jamuna with narrow standard errors may reflect the ongoing delineation. This corresponds to the verbal confirmation of the fish population decline by the local fishermen of the respective rivers. These findings suggest the importance of conservation strategies targeted at the genetic enrichment of vulnerable riverine populations.

Comparable studies in other freshwater systems, such as those on *Cyprinus carpio* and *Oncorhynchus mykiss* , have shown that populations with low N_e_ are more prone to inbreeding depression and reduced adaptive potential under environmental stress, reinforcing the relevance of our findings to broader conservation genetics (Christie et al. 2012; Frankham 2005; Wang et al. 2016). Moreover, recent studies highlight the global decline in genetic diversity across many species over the past three decades, emphasizing the urgency of conservation efforts to safeguard genetic health (Parreira et al. 2025; Shaw et al. 2025). Such measures can mitigate biodiversity loss (Langhammer et al. 2024), as maintaining an N_e_ above the critical threshold is vital for preserving genetic diversity and ensuring adaptability to environmental changes. Thus, strengthening conservation efforts in the Padma and Jamuna rivers, as well as Halda, is not only crucial for sustaining *L. rohita* population but also aligns with broader strategies for the genetic management of freshwater fish biodiversity under increasing anthropogenic and climatic pressures.

The population genetic structure of *L. rohita* has been studied from three riverorigins and one assemblage of cultured fishes of Bangladesh, focusing on a region of the *cytb* gene, like previous such reports (Habib and Vindhya 2011). However, our study indicates low genetic variability of *cytb* as a marker. So, necessary caution should be taken before generalizing the present analysis. Also, this result suggests that the *cytb* fragment analyzed may not have sufficient resolution to capture finer‐scale population differentiation. Therefore, it would be beneficial to include additional mtDNA and nuclear gene regions (i.e., fast evolving microsatellites) in such a study to understand the population better.

In our discussion about the differences in conservation strategies among rivers, we have observed a potential relationship between conservation policy efforts and variations in genetic diversity. While this correlation does not necessarily prove a cause‐and‐effect relationship, it stresses the importance of implementing stricter conservation strategies to protect fish and other species. Furthermore, our work suggests the need to enhance the genetic diversity of less diverse subpopulations, such as those in the Padma and Jamuna rivers, through controlled fishing practices, reintroduction, and other measures. Additionally, there is a need to develop commercial, allele‐specific molecular markers for measuring habitat‐specific differences and supporting selective breeding programs. Conducting further studies on the genetic status and diversity of *L. rohita* across more areas will provide crucial data for improving conservation efforts and biodiversity management. This study can be extended to other fish species as well.

## Author Contributions

**Md. Mahfuzur Rahman:** data curation (equal), formal analysis (equal), investigation (equal), visualization (equal), writing – original draft (equal). **Khandaker Asif Ahmed:** formal analysis (equal), methodology (equal), visualization (equal), writing – review and editing (equal). **Md. Golam Rabbane:** investigation (equal), project administration (supporting), writing – review and editing (equal). **Mohammad Shamimul Alam:** conceptualization (lead), data curation (equal), funding acquisition (lead), investigation (equal), methodology (equal), project administration (lead), supervision (lead), writing – review and editing (equal).

## Disclosure

Benefit‐Sharing Statement: Benefits from this research accrue from the sharing of our data and results on public databases as described above.

## Ethics Statement

No ethics approval was required for the current study.

## Conflicts of Interest

The authors declare no conflicts of interest.

## Supporting information


File S1.

File S2.

File S3.

## Data Availability

Unique haplotype data are deposited to NCBI Nucleotide Database (Accession numbers PQ458546 ‐PQ458556).
