# Supplementary material for: Genetic Diversity Resonates With Conservation Strategies: A Case Study of Labeo rohita Population
Source: Ecol Evol. 2025 May 25;15(5):e71480. doi: 10.1002/ece3.71480 (PMC12103948; doi:10.1002/ece3.71480)
Supplement: Supplementary file 1 — File S1. [file ECE3-15-e71480-s001.docx]

**Supplementary Table 01:** Percentage of different haplotypes among the populations of *L. rohita* in Bangladesh.

| **Haplotypes** | **Accession No.** | **Total (%)** | **Culture (%)** | **Halda (%)** | **Jamuna (%)** | **Padma (%)** |
| --- | --- | --- | --- | --- | --- | --- |
| Hap1BD | PQ458546 | 78 | 66 | 72 | 86 | 88 |
| Hap2BD | PQ458547 | 1.5 | 0 | 0 | 0 | 5 |
| Hap3BD | PQ458548 | 3 | 0 | 0 | 3 | 8 |
| Hap4BD | PQ458549 | 0.72 | 0 | 0 | 3 | 0 |
| Hap5BD | PQ458550 | 0.72 | 0 | 0 | 3 | 0 |
| Hap6BD | PQ458551 | 4.5 | 0 | 16 | 3 | 0 |
| Hap7BD | PQ458552 | 5.1 | 21 | 0 | 3 | 0 |
| Hap8BD | PQ458553 | 3.64 | 0 | 13 | 0 | 0 |
| Hap9BD | PQ458554 | 0.72 | 3 | 0 | 0 | 0 |
| Hap10BD | PQ458555 | 0.72 | 3 | 0 | 0 | 0 |
| Hap11BD | PQ458556 | 1.6 | 7 | 0 | 0 | 0 |
