# Supplementary material for: Genetic Diversity Resonates With Conservation Strategies: A Case Study of Labeo rohita Population
Source: Ecol Evol. 2025 May 25;15(5):e71480. doi: 10.1002/ece3.71480 (PMC12103948; doi:10.1002/ece3.71480)
Supplement: Supplementary file 3 — File S3. [file ECE3-15-e71480-s002.docx]

**Supplementary file: Genetic diversity resonates with conservation strategies: A case study of *Labeo rohita* populations**

Running Title: Genetic diversity in *L. rohita* populations

Md. Mahfuzur Rahman^1^, Khandaker Asif Ahmed^1,2^, Md. Golam Rabbane^3^, Mohammad Shamimul Alam^1*^

**Authors Affiliations:**

^1^Genetics and Molecular Biology Laboratory, Department of Zoology, University of Dhaka, Dhaka-1000, Bangladesh.

^2^CSIRO Australian Animal Health Laboratory, East Geelong, VIC 3220, Australia.

^3^Fisheries Genetics and Biotechnology Laboratory, Department of Fisheries, University of Dhaka, Dhaka-1000, Bangladesh.

**Corresponding Author Email Address:** [shamimul@du.ac.bd](mailto:shamimul@du.ac.bd)

**Supplementary fig. S1: Estimation of potential genetic lineage number (a) with- and (b) without- spatial modelling.**


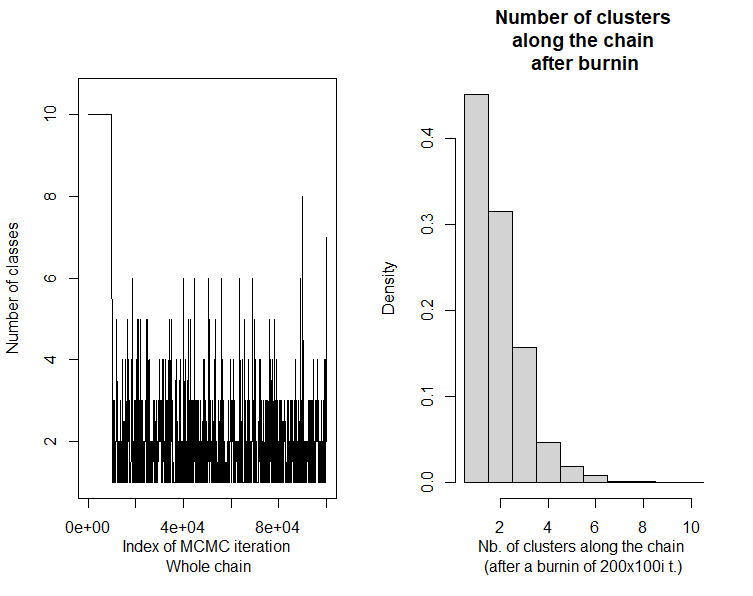

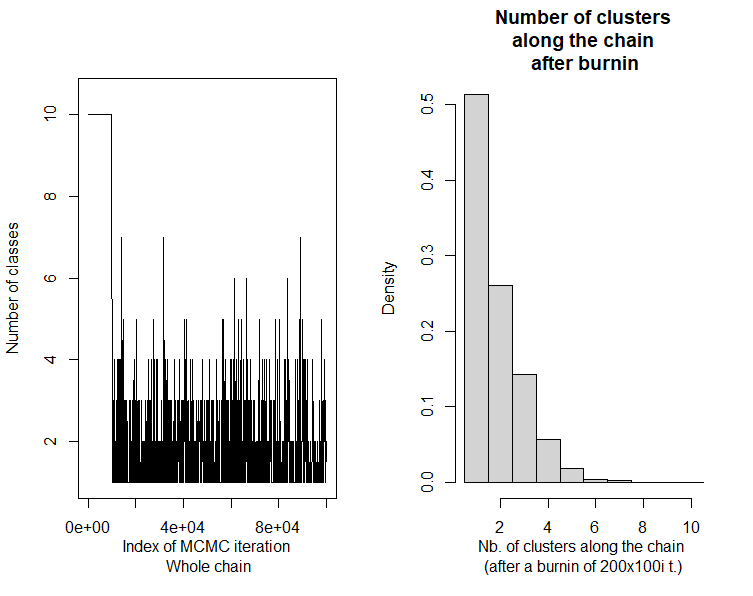


1. (b)
